# The social phenomenon of rare diseases: a logical-modal analysis

**DOI:** 10.3389/fsoc.2026.1810602

**Published:** 2026-07-13

**Authors:** Enrique Fernández-Vilas, Juan R. Coca, Nicolás Plaza-Gómez, Fernando Soler Toscano

**Affiliations:** 1 University of Valladolid, Soria, Spain; 2 University of Salamanca, Salamanca, Spain; 3 University of Seville, Seville, Spain

**Keywords:** Durkheim, institutionalization, modal logic, policies of recognition, rare diseases, social facts

## Abstract

This paper explores the social phenomenon of rare diseases through a logical-modal analysis. Although rare diseases originate within a biomedical framework and are increasingly recognised by institutional and empirical literature as issues of equity, inclusion, care and participation, this recognition has not usually been formalised in Durkheimian terms. The study applies modal logic, based on possible-worlds semantics and Kripke models, to clarify the conditions under which rare diseases acquire the status of social facts. By modelling behavioural change, coercion, generality and existence independent of individual manifestations, it shows how rare diseases transcend isolated clinical cases and impose structured constraints on social roles, families, institutions and policies. Empirical illustrations from international policy, employment studies, caregiver research and participation surveys are used to anchor the modal variables without treating the model as a statistical validation. The analysis argues that the transition from fragmented recognition to consolidated social fact depends on collective action, institutionalisation and normative recognition. The formalisation identifies intermediate configurations and a stabilised threshold state in which rare diseases become durable and socially binding within the model.

## 1 Introduction

Rare diseases, despite their low prevalence, constitute a major challenge for health systems and for social structures more broadly. In Europe they are commonly defined as conditions affecting fewer than 5 persons per 10,000 inhabitants, yet taken together they involve millions of people worldwide. This scale, together with their clinical complexity and social consequences, calls for an approach that brings biomedical and sociological perspectives into the same analytical frame. In this setting, Durkheim’s concept of the social fact offers a suitable lens for understanding how rare diseases exceed individual cases and emerge as a collective phenomenon with economic, political, moral and cultural dimensions.

The aim of this paper is to examine rare diseases from a sociological standpoint using modal–logical analysis, drawing on possible-worlds semantics. The analysis seeks to show that, although rare diseases originate as biomedical entities, they can be conceptualised as social facts in Durkheim’s sense, since they display properties of coercion, externality and generality that tie them to social structures and collective processes.

At the same time, the argument developed here does not assume that rare diseases lack social recognition. Recent institutional and empirical work already treats them as issues of equity, social inclusion, disability, employment, caregiving and access to rights. The World World Health Organization’s 2025 resolution on rare diseases recognises barriers to full and effective participation in society, discrimination, psychosocial consequences and vulnerability across education, employment, financial well-being and leisure (World Health Organization 2025). Systematic reviews and large-scale survey evidence similarly document work impairment, caregiver burden, unmet support needs and restrictions on school, work and community participation (Bougas et al., 2025; Atkins and Padgett, 2024; Mcmullan et al., 2022; Faye et al., 2025). The problem addressed in this article is therefore narrower: how can this empirical and institutional recognition be formalised as a transition from contingent visibility to a Durkheimian social fact?

The use of modal logic in this analysis is motivated by the need to clarify the conditions under which a phenomenon that originates in a biomedical domain acquires the status of a social fact in the Durkheimian sense. Existing approaches in the sociology of health have extensively documented patient experiences, forms of collective mobilisation and institutional responses associated with rare diseases. However, these analyses have largely focused on describing observed developments and their historical sequencing. Less attention has been paid to specifying the logical conditions that structure the transition from fragmented and episodic forms of recognition to configurations in which rare diseases exert stable, generalised and socially binding constraints on individuals and institutions.

By modelling social configurations as possible worlds and social transformations as accessibility relations between them, modal logic makes it possible to examine not only how recognition has developed historically, but also which combinations of coercion, externality and generality are sufficient to stabilise that recognition across plausible social contexts. This framework allows for the identification of intermediate configurations in which rare diseases display some, but not all, of the properties required of a social fact, as well as for the specification of threshold states in which those properties become jointly and necessarily realised. In this way, rarity is treated not as a purely statistical attribute, but as a relational and diachronic feature whose sociological significance depends on the modal structure of the social environments in which rare diseases are embedded.

The modal framework offers a formal specification of an issue already implicit in Durkheim’s definition of the social fact, namely the conditions under which a set of practices, representations and constraints acquires an existence independent of individual manifestations and maintains its efficacy across changing contexts of awareness and policy. When applied to rare diseases, this approach allows for a systematic analysis of the transition from statistical invisibility and social dispersion to institutional consolidation, and for the identification of the conditions under which a health-related phenomenon attains a durable and socially necessary status.

The study investigates the way in which rare diseases move from an isolated health problem to an issue of social concern that demands attention from health systems and policymakers. Modal logic is employed to model the social transformations through which rare diseases come to be recognised as a social phenomenon that cannot be reduced to individual manifestations. The central thesis is that these conditions affect those who live with them and, through their repercussions on families, organisations and institutions, influence society. This influence contributes to policy change, to the incorporation of patients with rare diseases into health systems on specific terms and to the eventual integration of rare diseases into wider political and social agendas.

The analysis assumes that the transformation of rare diseases into a social fact follows a gradual trajectory shaped by social, political and institutional factors. This trajectory can be traced from phases of invisibility and fragmentation to a situation in which rare diseases acquire the status of a consolidated public concern. In that process, the emergence of rare diseases as a stable social fact reflects both their social dimension and the role of collective action and mobilisation in reshaping normative and institutional frameworks.

The article is situated at the intersection of sociology of health, Durkheimian theory of the social fact and formal approaches to social change. The claim is not that rare diseases have never been treated as social problems or as socially consequential conditions. Rather, the contribution lies in specifying the conditions under which such recognition ceases to be episodic, descriptive or policy-specific and becomes a Durkheimian social fact, that is, a configuration of external, coercive and general constraints that persists across plausible social transformations. Existing work has tended to focus on patient experience, social representations, activism, health inequities and politics of recognition, while formal frameworks capable of specifying the modal threshold at which a biomedical phenomenon qualifies as a social fact remain scarce. The objective here is therefore to develop a formal criterion for the Durkheimian social fact that makes explicit the minimal conditions of behavioural change, coercion, externality and generality required for such a claim to hold.

The article makes three main contributions. First, it reformulates the concept of social fact as a set of properties that can be operationalised through propositional variables (m, c, g, e) and evaluated across different historical and social contexts. Second, it introduces Kripkean possible-worlds semantics as a tool for modelling social change, and shows how processes of institutionalisation, collective action and normative recognition can be represented as accessibility relations between social states. Third, it argues that the consolidation of rare diseases as a social fact can be understood as the modal stabilisation of specific properties in a necessary state of the model (w16), which provides a formal account of the transition from invisibility and fragmentation to incorporation into public agendas and systems of social protection.

The use of modal logic in this framework gives additional explanatory leverage by bringing into focus possible trajectories of social transformation, critical thresholds and points of no return at which rare diseases cease to appear as a collection of isolated medical cases and acquire the status of a fully constituted Durkheimian social fact.

## 2 Background: on the concept of social fact

In the late nineteenth and early twentieth centuries, scientific and philosophical thought was strongly shaped by positivism. Within sociology, functionalism and conflict theories sought to explain social structure and dynamics at the macro level, while symbolic interactionism and interpretive sociology shifted attention towards individual interpretations and everyday interactions. Durkheim developed the concept of the social fact within this positivist horizon. His approach employs a hypothetico–deductive framework to test *a priori* hypotheses, typically formulated in quantitative terms, from which functional relations between causal and explanatory factors and outcomes can be derived (Park et al., 2020). In this perspective, social research is grounded in empirical standards, with the social fact as its central unit of analysis (Musa, 2024). It is a sociological realist conception, in which society is described as a whole that exceeds the sum of individuals, and social facts exist independently of particular persons. Durkheimian sociology thus takes as its object the investigation of social forces, crystallised in moral norms that, through specific sequences, become social facts. In a similar vein, the American Sociological Association defines sociology as the discipline that studies social life, social change and the social causes and consequences of human behaviour.

Comte (1896) already argued that a sociological methodology, with its focus on social conditions and processes, should represent a significant advance in the control of disease. This intuition underlines the need to approach health and illness as social realities, a point already presents in the classical tradition. More recently, and drawing on Durkheim’s concern with norms, structures and social processes, Parsons identified the doctor–patient relationship as a paradigmatic element of the social system (Bradby, 2012). Social problems related to health can be described as objective and observable situations with negative social repercussions, affecting collectives and presenting at least a potential social aetiology. A social problem emerges from interaction and social circumstances and provides a useful vantage point for understanding health and illness (Solanki and Mittal, 2024). In Mauss (2016) terms, the relation between social fact and disease can be clarified through the example of the COVID-19 pandemic, which constitutes a “total social fact” in the sense that it affects multiple dimensions of social life simultaneously (Labora González and Fernández-Vilas, 2024; Ghahramani et al., 2021).

Rare diseases, therefore, should not be described as lacking all social recognition. They increasingly meet the criteria of a social problem and are addressed in institutional, clinical, advocacy and empirical literatures. However, this recognition remains uneven, fragmented and often more descriptive or policy-oriented than formally theorised as a Durkheimian social fact. The category of rare disease has an epidemiological origin: in Europe, it refers to conditions that affect fewer than 5 persons per 10,000 inhabitants. Each condition may remain statistically inconspicuous and diagnostically dispersed, but the category as a whole generates consequences across economic, political, moral, legal and care-related dimensions of social life. Dispersion and low prevalence can hinder public awareness and coordinated response, while institutional categorisation, patient mobilisation, disability recognition and policy agendas increasingly disclose their aggregate social impact. The relevant theoretical question is thus not whether rare diseases have any social dimension, but under what conditions the accumulation of coercive practices, external institutional categories and generalised consequences reaches the threshold of a social fact.

Current international estimates indicate that more than 300 million people live with a rare disease worldwide, with the majority of known rare diseases beginning in childhood (World Health Organization 2025). When the effects on family members and informal caregivers are included, the social perimeter of rare diseases extends well beyond diagnosed individuals. This extension is important for the present argument because it shows that the category operates not only through clinical prevalence, but also through the organisation of care, labour-market participation, social support, access to services and political recognition.

A large proportion of rare diseases entail some degree of physical, intellectual or sensory disability. The WHO (2025) assesses that rare diseases are associated with disability. Recognising disability as a universal social fact helps to illuminate the cultural specificities of persons with impairments and to reflect on the unstable boundaries of human nature (Ginsburg and Rapp, 2020). This provides an additional reason to examine whether rare diseases themselves can be treated as a social fact.

In The Rules of Sociological Method, Durkheim (1982) defines a social fact as any way of acting, thinking or feeling that is external to the individual and capable of exercising constraint upon them. He identifies three key features of social facts. First, coercion: social facts exert pressure on individuals, shaping their conduct in line with social norms and expectations. Second, externality: social facts exist outside the individual and arise from society. Third, generality: social facts are collective and recurrent, spreading through the whole of society or a significant part of it.

Durkheim (1982) maintains that social facts constitute a distinct class of phenomena to which the qualifier “social” properly applies. They differ from natural phenomena and from strictly individual phenomena such as a personal feeling or emotion, although social forces may mediate the latter. Social facts cannot be assimilated to organic phenomena, since they consist of practices and representations, nor to psychological phenomena, which exist only within and through individual consciousness. They are objective realities that do not derive from the biological or psychological constitution of the persons involved (Durkheim, 1982). Suicide provides the classical example (Durkheim, 2005): beyond its psychological dimension, it appears as a phenomenon influenced by social conditions. Social facts are distributed throughout society or across a substantial part of it and can be observed with statistical regularity. Social problems such as poverty, crime or, in the present case, health, affect large portions of the social structure and thereby differ from problems of a purely personal character (Solanki and Mittal, 2024). A pattern of collective conduct that characterises a given society and that possesses an existence of its own, independent of individual manifestations, qualifies as a social fact. Durkheim (1982) notes that statistical regularity does not cause the social character of the fact; it is instead a sign of its collective nature.

For Durkheim (1982), the thoughts that occupy individual consciousnesses and the movements that many people repeat do not count as social facts simply because they are widespread. Earlier attempts to define social facts on this basis confuse them with their individual “incarnations.” What matters for sociological analysis are the beliefs, tendencies and practices of the group considered as a whole.

Practices, beliefs, values and moral norms that guide conduct and constitute social facts are determined by a plural subject, a collective consciousness composed of many individual consciousnesses. Moral life emerges from interaction between individuals and from this collective consciousness (Musa, 2024). The condition of plurality is preserved because the distinctive character of the constituent members is maintained (Walsh 2020). Acts can be regarded as practical tests of the capacity of the plural subject to generate obligations or shared values. These acts, as events that in themselves express social obligation, may be understood as a specific kind of evidence for the plural subject; their social character can nonetheless be overlooked if the values and obligations they encode are not considered (Bojanic and Cvejic, 2018). The type of social facts considered here arise from institutions and cultural practices that orient individual conduct and from constraints produced by limited possibilities of choice.

Social facts can therefore involve two kinds of constraint: constraints that stem from the absence of alternatives and constraints that require choice to be exercised in accordance with established ideas. Durkheim drew a clear distinction between pressure in the physical domain and pressure in the social domain (Padovan, 2015). The former operates through morphological factors, whereas the latter relies on collective representations. Physical and moral environments differ, and the pressure exerted by one or several bodies upon others should not be confused with the pressure exerted by the group consciousness upon the consciousness of its members (Durkheim, 1982). At the group level, mental products such as knowledge, forms of expertise and systems of meaning correspond to shared visions that are common to a social formation and transmitted through communication (Kalampalikis and Apostolidis, 2021).

Against this backdrop, the study advances the following hypotheses:

*H_1_*. Although rare diseases are defined as a biomedical concept, they possess a social correlate that qualifies them as a social fact.

*H_2_*. The concept of social fact can be operationalised logically, which facilitates its use in social analysis.

To examine these hypotheses, the study pursues the following objective:

*O_1_*. To investigate, by means of modal logic, Durkheim’s definition in relation to rare diseases from a social perspective.

Modal logic makes it possible to formalise processes, assertions and relations and to analyse whether specific configurations hold across a range of possible worlds. The choice of Kripkean modal–temporal logic as a framework for examining whether the notion of “rare disease” constitutes a social fact responds to this capacity. A conventional sociological gaze might overlook rare diseases as a significant social fact because of their low point prevalence and asynchronous patterns of manifestation. Kripkean semantics, by contrast, invites enquiry into what a phenomenon is, what it could be and what it has been across different possible worlds and temporal moments. Within this framework, “rarity” is approached as a relational and diachronic construct whose truth-conditions depend on a historically, socially and normatively structured context. The approach suggests that collective impact, potential social aetiology and systemic repercussions function as necessary properties that are progressively actualised over time. On this basis, even if public visibility is intermittent, the status of rare diseases as a social fact can be argued for as a necessary and demonstrable truth within the model.

## 3 Methodology

### 3.1 Study design

The article adopts a theoretical–methodological design based on modal–logical analysis applied to a substantive problem in the sociology of health: the treatment of rare diseases as social facts in the Durkheimian sense. The analysis draws on an analytical review of sociological and philosophical literature on social facts and on rare diseases, together with relevant institutional and regulatory documents (national plans, specific legislation and position papers issued by international bodies and patient organisations). This material is used to identify the key dimensions of coercion, externality and generality at work in this field and to delimit the range of configurations that are sociologically plausible.

On this theoretical and empirical basis, a Kripke model M = ⟨W, R, V⟩ is constructed, where each possible world w ∈ W represents an ideal-typical social configuration of rare diseases. Each configuration is characterised by a combination of four properties: changes in individual behaviour (m), external coercion exerted on individuals (c), social generality (g) and existence independent of individual manifestations (e). The accessibility relation R is interpreted as the set of plausible social transformations, such as the creation of associations, legal reforms, expansion of coverage or shifts in collective awareness. The valuation function V assigns to each propositional variable the set of worlds in which it holds, in accordance with the way these properties appear in the literature and in institutional documentation.

Methodologically, the procedure unfolds in three steps. First, Durkheim’s definition of social fact is operationalised in terms of propositional variables. This step is grounded in the literature and in available evidence to justify that these variables capture empirically observable dimensions in the field of rare diseases, for example the presence of coercion through diagnostic norms, access criteria or control mechanisms, the widespread recognition of rare diseases as a public issue, or their translation into relatively stable institutional categories. Second, an accessibility structure is defined that represents non-reversible trajectories of social change, consistent with the historicity of institutionalisation processes and with the cumulative character of social rights. The relation R is constrained so that cycles implying regression from higher to lower levels of recognition do not occur within the model. This structure leads to a stabilised state (w16) in which rare diseases qualify as a social fact in a necessary way. Third, modal formulae are derived and examined that express sociologically relevant properties of the process, such as the possibility of reaching a state of necessary recognition, the maximum length of transformation chains or the conditions under which a problem remains invisible.

This approach is oriented towards providing a formally consistent and empirically informed reconstruction of the process through which rare diseases move from a dispersed set of clinical cases to consolidation as a social fact. It does not aim to generate statistical estimates or direct empirical generalisations. The model is assessed in terms of internal logical coherence and sociological plausibility. The trajectories allowed by the accessibility relation and the necessary properties of the final state are required to align both with Durkheimian theory of social facts and with the historical evolution of policies, associative movements and regulatory frameworks around rare diseases.

### 3.2 Empirical anchoring of the modal variables

The modal model is formal and ideal-typical, but its variables are anchored in concrete empirical and institutional materials. These materials are not used as a full case study or as a statistical test of the model. They function as illustrations showing how the propositions m, c, g and e can be interpreted in observable contexts. Table 1 summarises the empirical anchoring used in the revised analysis.

**Table 1:** Empirical anchoring of the modal variables.

Empirical evidence/case	Modal interpretation
Institutional recognition: the World Health Assembly resolution on rare diseases recognises barriers to full and effective social participation, discrimination, psychosocial consequences, vulnerability across education, employment, financial well-being and leisure, and the need for integrated health, social and community services (World Health Assembly 2025).	Supports externality (e) and generality (g), because rare diseases are treated as a public category requiring coordinated institutional action; it also supports coercion (c) insofar as barriers and eligibility systems restrict life chances.
Work participation: a systematic review of 44 studies found that individuals with rare diseases were less likely to be employed or more likely unemployed in 68% of the studies, more likely to be work disabled in 87%, had more missed work time in 90%, and more perceived impairment at work in 100% (Bougas et al., 2025).	Anchors behavioural modification (m), coercion (c) and generality (g), because the disease category modifies trajectories of employment, productivity and social integration beyond individual clinical episodes.
Family and caregiving: systematic, scoping and rapid reviews report psychological distress, lower quality of life, caregiver burden, changes in social support, unmet financial, social, psychological and information needs, and barriers to services (Atkins and Padgett, 2024; Černe et al., 2024; Mcmullan et al., 2022).	Anchors m, c and g by showing that rare diseases restructure family roles and informal care systems and impose constraints on non-patient actors.
Participation and disability: the Rare Barometer survey measured independent living and social participation and reported high disability rates, difficulties obtaining public support, discrimination, unemployment or inability to work, and limited school and community participation (Faye et al., 2025).	Anchors m, c, g and e: the category produces patterned restrictions across education, work and community life and becomes legible through collective measurement and advocacy.
Philosophy of medicine: recent work rejects a simple naturalism/normativism dichotomy and analyses how disease categories may be biologically real while involving social thresholds, values, epistemic marginalisation and institutional classification (Conley and Glackin, 2021; Thorell, 2024; Tosas, 2025).	Clarifies that the model does not deny biomedical reality; it formalises the social conditions under which a biomedical category becomes a Durkheimian social fact.

This anchoring also clarifies the epistemic status of the formal exercise. The model does not infer the Durkheimian status of rare diseases from any single dataset. It reconstructs the conditions under which recurring empirical patterns and institutional classifications can be treated as the components of a social fact.

### 3.3 Modal logic and Kripke models

A modal–logical analysis is carried out in order to determine the truth conditions for the claim that rare diseases are a social fact. Modal logic relies on the semantics of possible worlds developed by Saul Kripke (1963), with precedents in Leibniz’s *Monadology* and, more recently, in Lewis (1986) On the Plurality of Worlds, among others. The central idea is that of possible worlds, which provides a framework for analysing notions such as counterfactuality (reasoning about what could happen or could have happened in a given situation) and modality (possibility and necessity). Kripke (1959, 1963) developed a formal semantics for modal logic whose models have the form M = ⟨W, R, V⟩ and are defined by three components.

W is a non-empty set of possible worlds or states, which represent the situations that may obtain. Van Bethem (2010) emphasises the variety of interpretations available for possible worlds w, from metaphysical scenarios to computational states, and including domains such as science fiction, chess or card games. The appropriate interpretation depends on the kind of modal logic employed and on the object of study. In the present framework, possible worlds are interpreted as social situations. R is a binary accessibility relation on W. If the ordered pair (w_1_, w_2_) belongs to R, then the state w_2_ is accessible from w_1_. In this analysis, accessibility encodes the social transformations that may occur from a given state. If (w_1_, w_2_) and (w_1_, w_3_) belong to R, this indicates that the social context represented by w_1_ can evolve into either w_2_ or w_3_. Finally, V is a valuation function that determines in which worlds each propositional variable p is true. If the proposition m represents that rare diseases produce changes in people’s behaviour, then V(m) contains the states in which this condition is satisfied.

Given a Kripke model M = ⟨W, R, V⟩ and a world w ∈ W, the truth value of formulae constructed from a set P of propositional variables (representing facts that may or may not obtain), propositional operators ¬ (negation) and ∧ (conjunction), and modal operators □ (necessity) and ◊ (possibility) can be evaluated. For any formula *φ*, the following clauses apply:

- M, w ⊨ *φ* if and only if w ∈ V(*φ*), in the case where *φ* ∈ P. A propositional variable *φ* (or atomic proposition) is true at world w in model M when w belongs to V(*φ*).
- M, w ⊨ ¬*φ* if and only if it is not the case that M, w ⊨ *φ*. Negation is true when the formula to which it applies is false.
- M, w ⊨ *φ* ∧ *ψ* if and only if M, w ⊨ *φ* and M, w ⊨ ψ. Conjunction requires both conjuncts to be true.
- M, w ⊨ □*φ* if and only if, for every world v ∈ W such that (w, v) ∈ R, M, v ⊨ *φ*. The formula □*φ* expresses that *φ* is necessary; its truth at w requires that *φ* hold in all states accessible from w.
- M, w ⊨ ◊*φ* if and only if there exists a world v ∈ W such that (w, v) ∈ R and M, v ⊨ *φ*. The formula ◊*φ* expresses that *φ* is possible; its truth at w requires the existence of at least one accessible world v where *φ* is true.

If further operators such as ∨ (disjunction), → (conditional) or ↔ (biconditional) are introduced, their truth conditions coincide with those of propositional logic: *φ* ∨ ψ is equivalent to ¬(¬*φ* ∧ ¬ψ); *φ* → ψ is equivalent to ¬*φ* ∨ ψ; and *φ* ↔ ψ is equivalent to (*φ* ∧ ψ) ∨ (¬*φ* ∧ ¬ψ).

These clauses define the truth of a formula at a world w of a model M. If M, w ⊨ *φ* holds for every w ∈ W, then *φ* is said to be valid in M, that is, it holds in all worlds of the model, and this is denoted by M ⊨ *φ*. If, for every model M that can be defined, M ⊨ *φ* holds, then one writes ⊨ *φ*, and *φ* is a tautology of modal logic.

## 4 Results

**Figure 1:**
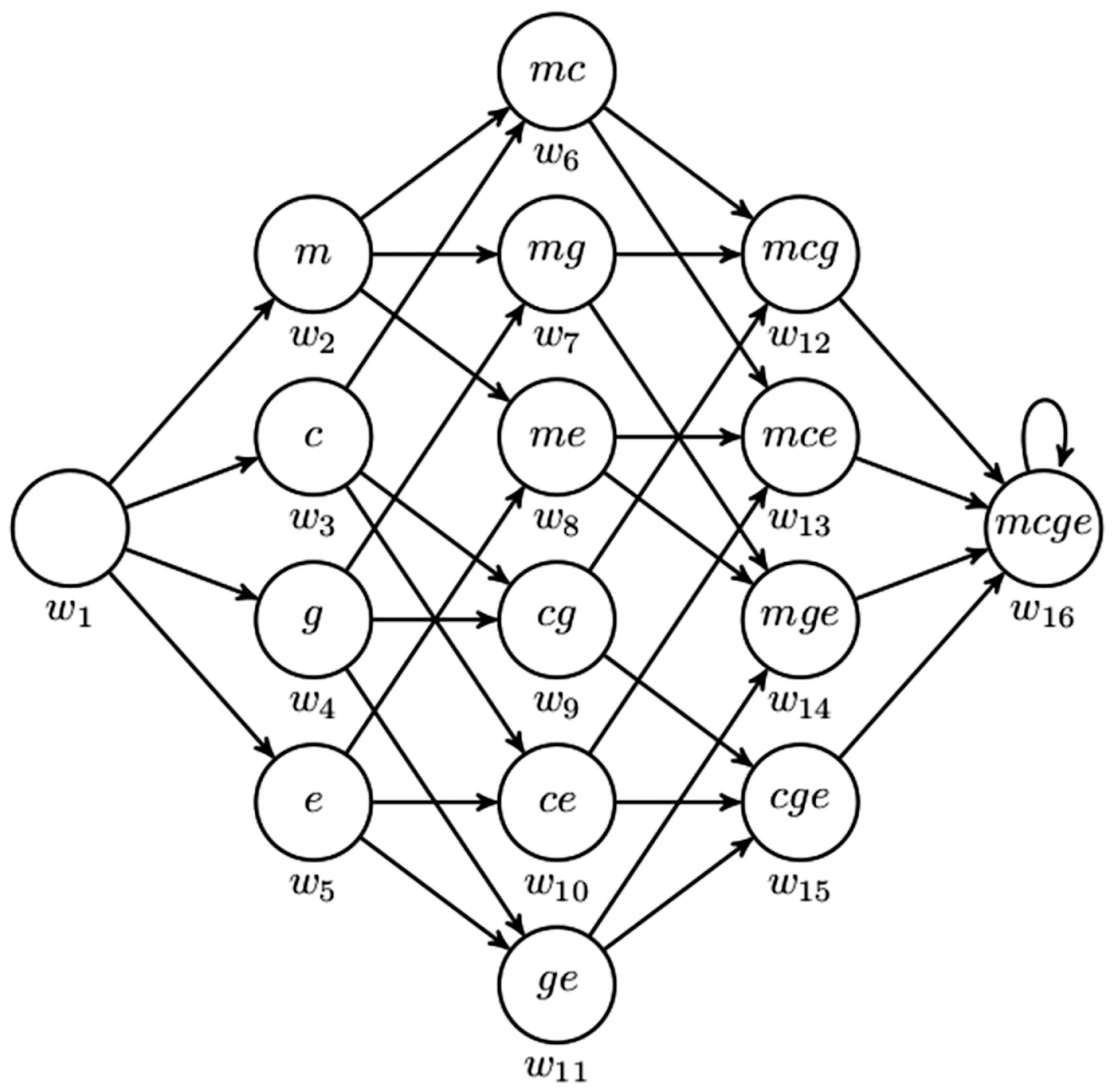
Kripke model with propositional variables. Source: own elaboration.

We take M to be the Kripke model represented in Figure 1. The model uses the propositional variables m, c, g and e to represent that rare diseases

- Produce changes in people’s behaviour: m.
- Exert external coercion on individuals: c.
- Display social generality: g.
- Exist in their own right, independently of individual manifestations: e.

In each state from w1 to w16, the propositional variables written inside the corresponding node are true; the remaining variables are false in that state. For example, in w6 we have

M,w6⊨m∧c∧¬g∧¬e

Each state represents a particular configuration of the social characteristics of rare diseases. Accessibility (the arrows between states) represents the social transformations that alter the status of rare diseases. The model assumes that these transformations are not reversible and that each of the four propositional variables can become true independently. If empirical or theoretical considerations ruled out certain combinations, the corresponding states would be removed from the model. For instance, if c could not occur without g, states w3, w6, w10 and w13 would not appear.

If social change were reversible, accessibility would also run backwards between some states. In the present model, the only reflexive arrow is at w16. This loop expresses that further social changes may still occur once rare diseases have acquired the status of social fact and, at the same time, it avoids the anomaly that arises in modal logic when a world has no accessible successors, in which case every formula of the form □*φ* becomes vacuously true.

Following Durkheim’s criteria, the proposition that rare diseases are a social fact is expressed as

H≔m∧c∧g∧e

Several formulae hold in the model and carry sociological significance:

1. H → □H
  Once rare diseases satisfy all four properties of a social fact in a given state, they continue to do so in every state accessible from it. In sociological terms, the status of rare diseases as a social fact is stable with respect to further transformations encoded in R.
2. ◇◇◇◇□H
  From any initial state it is possible to reach a situation in which rare diseases have become a necessary social fact. The chain of four possibility operators reflects the maximum length of a transformation path from w1 to w16 in the model.
3. (m ∨ c ∨ g ∨ e) → ◇◇◇□H
  As soon as at least one of the Durkheimian properties holds (that is, in any state other than w1), at most three further transformations are required to arrive at a state in which H holds necessarily. Once rare diseases acquire any of the four dimensions in a given configuration, the path to full and stable recognition as a social fact becomes shorter.

These results show how the model captures both the gradual accumulation of Durkheimian properties and the presence of thresholds in the process by which rare diseases move from invisibility to consolidated social recognition. The use of modality also opens the way to distinguishing different patterns of translation of these configurations into social responses, for instance through preventive, compensatory or restorative policies, depending on the region of the model in which a given health system operates.

A further structural feature of the model is that, for every state w, there is at least one state w’ such that (w, w’) ∈ R. In the case of w16, the only accessible state is w16 itself. A relation R with this property is called serial. When R is serial, the modal system validates axiom D:

□φ→◇φ

Which states that everything necessary is also possible. In all cases, the model validates axiom K:

□(φ→ψ)→(□φ→□ψ)

Which characterises normal modal logics based on Kripke semantics. In the following sections, the accessibility relation R will be modified to impose additional constraints, generating new logical properties and allowing further formulae with sociological relevance to be derived.

### 4.1 System T

In system T, the model M is modified so that the accessibility relation R becomes reflexive. As in the case of w16 in the base model, every state wi now has an arrow to itself, so (wi, wi) ∈ R for all i. This setting represents the possibility that social change does not alter the status of rare diseases: a given configuration may persist over time without transition to a different one, either because institutional arrangements remain stable or because attempts at reform fail to take effect.

On a reflexive frame, modal logic validates axiom T:

□φ→φ

So anything necessary (true at all accessible states) is also true at the current state, since the current state is among its own accessible successors. All the formulae that held in the base model continue to hold, and additional statements become true. In particular:

1. ¬H → ◇¬H
  Whenever rare diseases are not a social fact in a given state, there is always at least one accessible state – possibly the very same state – in which they continue not to be a social fact. This expresses the inertia of configurations in which rare diseases remain below the threshold of full social recognition and shows that mere passage of time does not guarantee movement towards H.
2. □H → H
  If it is necessary that rare diseases are a social fact, then they are already a social fact in the current state. Once the model reaches a configuration in which the Durkheimian status of rare diseases is secured across all accessible futures, that status is realised in the present as well.

System T thus captures a situation in which both recognition and non-recognition may display stability. Rare diseases can remain trapped in configurations where ¬H persists, or they can enter configurations where H holds and is robust under further social transformations. The reflexive structure of R makes explicit that remaining in the current configuration is always one of the available possibilities.

### 4.2 System S4

In system S4, the accessibility relation R is further constrained to be transitive (in addition to reflexive). In terms of the base diagram, this corresponds to adding arrows that connect states which were previously only reachable by following two or more steps of accessibility. Concretely, if A is the set of propositional variables true at some state wi (A ⊆ {m, c, g, e}) and B is the set of propositional variables true at another state wj, then whenever A ⊆ B there is an arrow from wi to wj. Socially, this means that a configuration in which rare diseases already exhibit a certain subset of Durkheimian properties can move in a single step to any configuration in which those properties are preserved and additional ones are added.

On reflexive and transitive frames, modal logic validates axiom 4:

□φ→□□φ

Which is equivalent to:

◇◇φ→◇φ

The latter formulation indicates that if something is reachable in two steps, then it is already reachable in one. In the present context, a single social transformation may lead directly to a configuration that previously required several intermediate stages. As a consequence, new formulae become valid, such as:

1. ◇H
  From every state in the model, at least one accessible state exists in which rare diseases are a social fact. Even when none of the Durkheimian properties is fully realised in the current configuration, there is always a possible immediate transformation that leads to a configuration where they are.
2. ◇□H
  From every state, it is possible to reach – in a single step – a configuration in which rare diseases are necessarily a social fact, that is, a state from which all accessible configurations preserve H. The model thus encodes the idea that one decisive social transformation (for instance, a comprehensive policy package or a major institutional reform) can place rare diseases in a position of stable recognition.
3. □H ∨ ◇□H
  In every state, either rare diseases are already a necessary social fact, or there exists a single social transformation that turns them into one. Formally, the model leaves no configuration from which rare diseases are both contingently non-social and structurally unable to become a stable social fact.

System S4 therefore represents a scenario in which the path towards the consolidation of rare diseases as a social fact is significantly compressed. Once some Durkheimian properties are in place, further institutional, political or cultural changes can, in principle, produce rapid transitions to states of full and enduring recognition.

The results of the modal analysis do not consist in empirical generalisations, but in the identification of structural properties of the process through which rare diseases acquire the status of a social fact. First, the model makes it possible to distinguish analytically between configurations in which rare diseases are contingently recognised and those in which their recognition becomes necessary with respect to the space of plausible social transformations. This distinction clarifies a dimension of social facts that is often implicit in descriptive accounts of institutionalisation.

Second, the results identify a set of intermediate configurations in which some, but not all, of the Durkheimian properties of the social fact are realised. These configurations function as thresholds in the transition from invisibility and fragmentation to consolidated social recognition, showing that the emergence of a social fact is not a linear accumulation of visibility or policy measures, but a structured process governed by specific combinations of coercion, externality and generality.

Third, the modal structure of the model captures the stabilisation of the social fact once these properties are jointly realised. In the stabilised configuration, the status of rare diseases as a social fact is preserved across accessible social contexts, formalising the idea that certain forms of institutional recognition generate durable and socially binding constraints that resist reversal.

## 5 Discussion

This article makes three interrelated contributions to sociological theory and to the sociology of health. First, it advances a formal reconstruction of Durkheim’s concept of the social fact by specifying its core properties—coercion, externality and generality—as analytically independent dimensions that can be combined and evaluated across different social configurations. This move allows the notion of social fact to be treated not only as a classificatory concept, but as a structured set of conditions whose joint realisation can be examined systematically. The contribution is therefore not the claim that rare diseases have never been recognised as socially consequential, but the formal specification of when that recognition becomes stabilised as a Durkheimian social fact.

Second, the article introduces modal logic as a methodological framework for the analysis of social change, conceptualising social configurations as possible worlds and institutional, political and cultural transformations as accessibility relations between them. This approach makes it possible to distinguish between contingent forms of recognition and configurations in which a phenomenon attains a necessary and stabilised social status, thereby addressing a dimension of social facts that remains implicit in most empirical and constructivist accounts.

Third, by applying this framework to the case of rare diseases, the article offers a novel sociological account of how phenomena characterised by low prevalence and fragmentation can nonetheless acquire the status of a social fact. The analysis shows that the social character of rare diseases does not depend solely on epidemiological aggregation or visibility, but on the modal consolidation of specific properties that render their recognition institutionally binding and socially unavoidable. In this sense, the article contributes both a formal analytical tool and a substantive reinterpretation of rare diseases as a durable and necessary social phenomenon.

The ensemble of individuals and their actions forms a social reality structured through social facts that condition the range of possible courses of action for each person. The concept of social fact, with its emphasis on structure, is frequently placed in tension with approaches centred on agency and rational calculation. Boudon (1977), Elster (2016) and Coleman (1994) have questioned more rigid structuralist accounts and have stressed that social dynamics require attention to interests, bounded rationality and conflicts between actors. From the standpoint of conflict theory, Coser (1967), Nisbet (1966) and Zeitlin (2000) have characterised Durkheimian sociology as conservative because of its focus on order and integration.

Giddens (1993) argues that Durkheim’s central concern is the changing nature of social order. Building on that reading, Giddens, Bhaskar (1979) and Alexander (1982) develop models that articulate relations between action and structure and attempt to move beyond a stark opposition between holist and individualist explanations. Greenwood (2003) underlines that this dispute calls for empirical testing and careful theoretical refinement; purely conceptual manoeuvres are insufficient.

External coercion as a defining feature of social facts appears at different levels and in varied contexts, ranging from small groups to large-scale formations. What matters is the way in which it is imposed generally upon individuals, regardless of personal will. Coleman’s (1994) “boat” offers a helpful device for connecting individual decisions with emergent macro-level outcomes and for depicting how micro-processes and social structures interlock.

Individuals act as producers of social forces and at the same time as subjects upon whom those forces bear. They participate in the creation and reproduction of structures and are shaped by norms, values and institutions. Decisions may have a strategic character, yet they are always taken within a background of shared beliefs and expectations. Olson (1965) drew attention to the tension between self-interest and collective action. Gintis (2003) argues that the internalisation of norms and values can be reconciled with the pursuit of individual advantage if rationality is understood in an extended sense that incorporates sanctions, reputation, social norms and processes of genetic–cultural coevolution. From this vantage point, values appear as dynamic social facts, undergoing transformation and transmission over time.

Marcucci (2022) maintains that sociology enables solidarity to be conceived as a social fact and as a precondition for modern politics. Organic solidarity, mediated by intermediary groups and associations, is especially relevant for understanding moral dependence in complex societies. Solidarity is not the central object of the present analysis, yet it resonates with the Durkheimian background and with the evolutionary view of social order that underpins it.

The examination of accessibility relations between possible worlds connects directly with these debates. Durkheim conceived social facts as anchored in specific contexts, in a “social world” that can be reached from a given situation. Pólos et al. (2010) insist on the need to exclude isolated worlds and to work explicitly with accessibility between possible worlds. The modal model developed here follows that line of argument. The analysis of the accessibility structure makes it possible to trace chains of distinct social configurations and to describe the conditions under which a stabilised state emerges.

The results indicate that rare diseases attain the status of social fact through a progressive and unidirectional process. This dynamic points towards a form of sociological gradualism in which social awareness is formed and transformed through intermediate worlds that act as thresholds and transitions between states of invisibility and states of recognition.

### 5.1 Theoretical and methodological contributions

At the theoretical level, the study reformulates the Durkheimian concept of social fact as a set of explicit properties – coercion, externality and generality – that can be represented by propositional variables. This move turns a mainly qualitative definition into a scheme that permits comparison between social configurations of rare diseases and clarifies in what sense they acquire the form of a social fact.

The use of Kripkean possible-worlds semantics provides a specific device for thinking about processes of social change. The accessibility relation is interpreted as a set of plausible transformations, including institutionalisation, collective action, regulatory reforms and shifts in collective consciousness. The model depicts chains of non-reversible social states in which certain properties, such as the recognition of rare diseases as a public problem, eventually become necessary.

The analysis also shows that the consolidation of rare diseases as a social fact can be read as the modal stabilisation of a final state of the model. In that state, the defining properties of the social fact hold with necessity. The trajectory from configurations marked by invisibility or fragmentation to a social world in which rare diseases appear as a fully established social fact is thus formalised through identifiable thresholds and points of no return.

On the methodological side, the proposal takes the form of a formal framework designed to interact with existing empirical approaches. The model helps to clarify assumptions, to compare scenarios and to explore in a systematic manner the logical implications of distinct institutional configurations. Modal logic is presented as an analytical complement for sociology of health and of rare diseases and is conceived as compatible with the qualitative and quantitative methods that dominate empirical work in the field.

### 5.2 The political dimension of the social fact

The recognition of rare diseases as a social fact involves a Durkheimian conceptualisation and, simultaneously, their inscription in political, institutional and normative structures that govern social life. Institutions occupy a central place in the transformation of a health problem into a socially acknowledged phenomenon through the formulation and implementation of public policies.

The institutionalisation of rare diseases has advanced through a combination of visibility, social pressure and collective action. The World Health Organization 2025’s 2025 resolution, the European Union and multiple patient associations have contributed to placing these diseases on the agenda as a matter of public health, equity and inclusion requiring governmental intervention (World Health Organization 2025). In the field of sectoral policies, the recognition of rare diseases as a social fact has given rise to specific strategic plans, such as the Spanish National Plan for Rare Diseases or the United States Orphan Drug Act of 1983, which has supported the development of treatments for low-prevalence conditions (Haffner et al., 2002). These initiatives seek to reduce inequalities in access to diagnosis, therapies, treatments and support, and constitute clear expressions of social recognition.

The trajectories encoded in the accessibility relation resonate with socio-historical analyses of the institutionalisation of rare diseases and the evolution of policy frameworks in European contexts (Coca et al., 2025), where successive regulatory reforms have progressively stabilised these conditions as a matter of public concern at both national and supranational levels.

Sociological debates have repeatedly addressed the relation between social facts and policy-making. Bourdieu (1994) argues that a problem becomes a matter of collective concern when actors capable of mobilising symbolic capital succeed in obtaining institutional recognition. In the area of rare diseases, patient movements and medical and scientific associations have acted as pressure groups that push states and international organisations towards legislation aimed at greater equality in health.

Questions of social justice emerge at the core of this political construction of the social fact. Inequalities in access to treatments and the high cost of many therapies have fostered regulatory frameworks oriented towards the inclusion of these patients within health systems. Fraser (2008a, 2008b) theory of justice emphasises the need to link redistribution and recognition, since the absence of recognition for particular collectives obstructs equitable access to basic rights and requires policies that address material and symbolic dimensions of injustice in tandem.

The modal accessibility model can also be read through this institutional lens. Each transition between possible worlds expresses a shift in the way society and the state recognise rare diseases and incorporate them into systems of social protection. Stabilisation at w16 suggests that, once a certain level of recognition has been achieved, institutions tend to develop mechanisms that reinforce visibility and sustained attention, in line with a consolidated politics of recognition.

### 5.3 The social fact of rare diseases through collective action

Rare diseases expose tensions between social norms and actual living conditions. Solanki and Mittal (2024) argue that, when community norms and values fail to engage with a social problem, the inability of society to safeguard the health of its members reveals a structural disconnection. The social burden of these conditions is visible in mortality, disability, years of life lost, hospital admissions and readmissions, long-term and palliative care needs and associated costs (Ferreira, 2019). Delaye et al. (2022) describe how these health problems restrict the social participation of those affected.

Olson (1965) showed that unorganised groups encounter substantial obstacles when trying to protect their shared interests. In the field of health, camaraderie and mutual support reduce feelings of abnormality and highlight the importance of support groups (Bane et al., 2005). Delisle et al. (2017) report that participation in such groups gives access to interaction with others in the same situation, information about disease and treatment, emotional support, open discussion, coping resources, a sense of empowerment and opportunities to advocate for improved care. Ashtari and Taylor (2023) show that patients frequently obtain through these networks information that is absent from formal health-care circuits. Associational life in this context can be interpreted as a rational form of cooperation and reciprocal solidarity, grounded in shared values and beliefs and oriented towards the maximisation of common interests through altruistic behaviour.

Rare diseases generate far-reaching social implications and contribute to shaping institutions and behaviours based on collective values, beliefs and practices. In modal terms, the transition towards w16 indicates that the recognition of rare diseases as a social fact stabilises in a particular possible world. The creation of support networks, which already appears in intermediate worlds such as w3 or w4, facilitates this process and suggests that social movements play a key role in transitions between different social configurations.

Individual experiences are transformed into collective force that seeks to modify perceptions and norms in a social environment inclined to homogenise and to isolate diversity. This tendency can produce negative identities and symbolic hierarchies between groups. Beyond their biological dimension, collective representations of rare diseases exert a strong influence on expectations and behaviour. Halley et al. (2023) emphasise that the demands of patients and families depend both on self-advocacy in relation to clinical care and on public advocacy directed towards research and equity. Lack of diagnosis (Labora González and Fernández-Vilas, 2022), uncertainty concerning treatments and prognoses (Quintal et al., 2024) and structural stigma linked to health, education, employment and accessibility (Munro et al., 2022) intensify these tensions.

Individual values rest on life histories and specific interpretative frameworks, which may be transformed through the experience of illness (Kleinman and Benson, 2006). Lack of information and treatments, the choice of specialists and decisions on novel therapeutic approaches produce shared situations of uncertainty that demand rational decision-making at both individual and collective levels. Actors and their organisations possess a relative capacity for transformative action and can come to embody social forces that crystallise as social facts. Through that action they generate shared values, common interests, norms, institutions and collective patterns of conduct. The experiences of adults living with rare diseases, with their profound moral and existential weight, call for changes in clinical practice and in the empowerment of patients (Quintal et al., 2023).

Advocacy organisations for rare diseases can shape research agendas and increase their relevance and quality (Berrios et al., 2024). These conditions have historically stimulated innovation and opened novel perspectives in biomedical and social research (Baynam et al., 2024). Within the European Union, political debate has incorporated measures designed to foster the development and availability of medicines for rare diseases as a group (French Ministry of Health, Youth and Sports, 2008). In recent years, communities and institutions have promoted research projects, public policies and development agendas that address the problem of rare diseases explicitly, taking seriously an aetiology that is both biological and social. This situation demands sustained collective commitment, since the social problems associated with these conditions exceed the capacities of isolated individual action.

### 5.4 Empirical illustrations and contemporary approaches

The empirical materials incorporated above show that rare diseases are already recognised in several contemporary sociological and policy domains. Employment research documents restrictions on work participation and work ability; caregiver research documents how rare diseases reorganise family life and informal support; participation surveys document barriers in school, employment, community life and independent living; and recent international policy frames rare diseases as a matter of equity and inclusion. These findings support the claim that rare diseases operate beyond the individual clinical case, but they do not by themselves establish the formal Durkheimian threshold analysed in the model.

This distinction is central for the novelty of the article. The model does not compete with empirical accounts of burden, participation, stigma, inequity or advocacy. It reorganises those accounts within a formal sociological question: under what conditions does a biomedical category become external to individuals, coercive in its effects, general in its social distribution and independent of particular manifestations? The added value of modal logic is to distinguish episodic recognition from stabilised recognition and to identify intermediate configurations in which some, but not all, of the Durkheimian properties are present.

The approach also connects with recent philosophy of medicine. Conley and Glackin (2021) argue that a naturalistic orientation to disease need not exclude a form of social constructivism. Thorell (2024) similarly challenges a strict opposition between naturalism and normativism and defends a more nuanced account of health and disease concepts. In the field of rare, poorly understood and underdiagnosed diseases, Tosas (2025) analyses epistemic injustice and argues that patients’ marginalisation can reveal how some medical taxonomies are also sociocultural constructions. These approaches are compatible with the present model because the model does not reduce rare diseases to social constructs; rather, it asks when biologically real conditions become organised as social facts through classification, institutionalisation, constraint and collective recognition.

### 5.5 Limitations of the model

The model has several limitations. First, modal necessity in this article is model-relative: it does not imply historical inevitability, metaphysical necessity or empirical irreversibility. It means that, given the accessibility relation defined in the model, once the relevant properties are jointly realised, all accessible successor states preserve the status of rare diseases as a social fact. Second, the model idealises social change as monotonic and non-reversible. This is analytically useful for representing cumulative institutionalisation, but it may understate policy retrenchment, welfare-state contraction, loss of public attention or unequal implementation across countries.

Third, the variables m, c, g, and e are treated as analytically independent, although empirical research may show dependencies among them. For example, institutional externality may be required before coercive access criteria can operate, or generality may depend on collective categorisation across heterogeneous diseases. Fourth, the empirical examples used here are illustrative and theory-anchoring; they do not constitute a statistical validation of the model. Fifth, the model treats rare diseases as a collective category, which is sociologically useful but may obscure differences among diseases, age groups, degrees of disability, diagnostic trajectories, national health systems and patient organisations. Finally, the framework is institution-sensitive insofar as several examples derive from European or international policy and advocacy contexts. Future work should test whether the same modal structure applies in low-resource settings and in contexts where patient organisations, registries and social protection systems are less developed.

## 6 Conclusion

The modal–logical analysis of the Durkheimian notion of social fact, applied to rare diseases, shows that conditions originating in a biomedical frame can acquire a social status that justifies their treatment as social facts in Durkheim’s sense. The analysis does not depend on the claim that rare diseases have lacked all social recognition. On the contrary, contemporary institutional and empirical evidence increasingly recognises them as issues of equity, inclusion, care, employment and participation. The specific contribution of the article is to formalise the conditions under which that recognition becomes external, coercive, general and stabilised across accessible social configurations.

From a modal perspective, the structure developed in the study captures the progressive transition through which rare diseases are consolidated as a social fact. The sequence of accessible worlds brings into view a temporally extended process that depends on social, political and institutional arrangements. The stabilised world in which the formulae expressing coercion, externality and generality hold with necessity can be interpreted as the point at which rare diseases are durably incorporated into public agendas and systems of social protection.

The analysis also highlights the contribution of collective action, associational life and support networks to this transformation. Patient organisations and allied actors participate in the movement from a health problem confined to clinical practice to a recognised matter of public concern. Legislative reforms, health policies and specific programmes addressing rare diseases appear in the model as transitions along the accessibility relation and as conditions that favour the emergence of a world in which rare diseases are endowed with institutional recognition.

The findings indicate that the social perception of a health condition is not governed by epidemiological prevalence alone. The impact on social structures, collective representations and organisational arrangements is decisive. The institutionalisation of rare diseases, driven by activism and by legal and regulatory recognition, contributes to their configuration as a stable social fact within the modal framework and can be related to observable processes of categorisation, policy design and organisational change.

At the same time, the status of rare diseases as a social fact remains exposed to ongoing dynamics of social change. Scientific and technological developments, shifts in health systems and broader political realignments can modify the space of accessible worlds and reposition rare diseases within it. Policies of recognition and redistribution directed at these conditions illustrate the link between social facts and processes of social justice and show how normative and material dimensions are intertwined in the construction of rare diseases as a collective concern.

This analysis remains at a formal and conceptual level and does not seek to offer a comprehensive empirical account of rare diseases, which can be developed in subsequent studies engaging directly with qualitative and quantitative data. The application of modal logic to the sociological conceptualisation of rare diseases thus provides a formal instrument for examining their transformation into a socially acknowledged phenomenon. The framework clarifies that the construction of a social fact involves objective constraints and patterned regularities, alongside interactional processes, normative codification and institutional embedding. It also clarifies the limits of the model: necessity is model-relative, the accessibility relation is idealised and the empirical examples are illustrative rather than confirmatory. The approach offers a basis for further work in which idealised modal structures are confronted with empirical materials on rare diseases, and for extensions to other areas of health and illness where the emergence and consolidation of social facts are at stake.

## Data Availability

The original contributions presented in the study are included in the article/supplementary material, further inquiries can be directed to the corresponding author.
